# Adopting Patient Portals in Hospitals: Qualitative Study

**DOI:** 10.2196/16921

**Published:** 2020-05-19

**Authors:** Pauline Hulter, Bettine Pluut, Christine Leenen-Brinkhuis, Marleen de Mul, Kees Ahaus, Anne Marie Weggelaar-Jansen

**Affiliations:** 1 Erasmus School of Health Policy & Management Erasmus University Rotterdam Rotterdam Netherlands; 2 Dutch Hospital Association Utrecht Netherlands; 3 Eindhoven University of Technology Eindhoven Netherlands

**Keywords:** patient portal, adoption, adoption processes, eHealth

## Abstract

**Background:**

Theoretical models help to explain or predict the adoption of electronic health (eHealth) technology and illustrate the complexity of the adoption process. These models provide insights into general factors that influence the use of eHealth technology. However, they do not give hospitals much actionable knowledge on how to facilitate the adoption process.

**Objective:**

Our study aims to provide insights into patient portal adoption processes among patients and hospital staff, including health care professionals (HCPs), managers, and administrative clerks. Studying the experiences and views of stakeholders answers the following question: How can hospitals encourage patients and HCPs to adopt a patient portal?

**Methods:**

We conducted 22 semistructured individual and group interviews (n=69) in 12 hospitals and four focus groups with members of national and seminational organizations and patient portal suppliers (n=53).

**Results:**

The effort hospitals put into adopting patient portals can be split into three themes. First, inform patients and HCPs about the portal. This communication strategy has four objectives: users should (1) know about the portal, (2) know how the portal works, (3) know that action on the portal is required, and (4) know where to find help with the portal. Second, embed the patient portal in the daily routine of HCPs and management. This involves three forms of support: (1) hospital policy, (2) management by monitoring the numbers, and (3) a structured implementation strategy that includes all staff of one department. Third, try to adjust the portal to meet patients’ needs to optimize user-friendliness in two ways: (1) use patients’ feedback and (2) focus on optimizing for patients with special needs (eg, low literacy and low digital skills).

**Conclusions:**

Asking stakeholders what they have learned from their efforts to stimulate patient portal use in hospitals elicited rich insights into the adoption process. These insights are missing in the theoretical models. Therefore, our findings help to translate the relatively abstract factors one finds in theoretical models to the everyday pragmatics of eHealth projects in hospitals.

## Introduction

Electronic health (eHealth) technology is generally considered promising for improving both the well-being and health of patients and the efficiency and effectiveness of the health care organization [1]. However, several studies show that its promise is not always fulfilled [1] and the results on the benefits gained are diverse [1,2]. This also applies to patient portals [2], which have sparked the interest of researchers, government policy makers, and health care organizations.

Previous studies define a patient portal in various ways [3-7]. Some consider them the same as a personal health record (PHR) [3]. Others regard them as a class of PHR [4]: whereas health care organizations own and control patient portals, PHRs are owned and controlled by patients themselves [5]. Grünloh et al [6] define patient portals as “provider-tethered applications that allow patients to access, but not to control, certain health care information (eg, their EHR [electronic health record]) and provide communication and administrative functions (eg, secure messaging, appointment booking, and prescription refill requests).” Wildenbos [7] adds the possibility of authorizing informal caregivers to share access to patient portals.

Despite its technological focus and aim, the success or failure of a patient portal relies on how it is used by patients and staff, such as health care professionals (HCPs), managers, and administrative clerks [1,2,8]. A systematic review shows often-limited use by patients and HCPs for seven nontechnical reasons [2]: (1) patients worry about the confidentiality of their personal health data, (2) patients are unaware of the portal, have no digital access, or think it will not be useful, (3) patients have low health literacy or find using the portal too complicated, (4) HCPs worry about increased workload and disruptions to their usual tasks, (5) HCPs lack the digital skills to interact with patients, (6) HCPs worry that they cannot respond fast enough to patients’ questions, and (7) HCPs are concerned that they can be held liable [2]. All seven reasons hinder the adoption of patient portals [2].

Researchers have developed theoretical models to explain or predict the adoption of eHealth technologies, including patient portals. Two systematic reviews on information technology adoption both mention three frequently used acceptance and adoption models [9,10]. Strikingly, these three general models are applied in all societal domains, not just health care [9]. Table 1 details these most-used models to facilitate an understanding of their ideas on the adoption process [9-20]. The table also includes the recently developed NASSS (nonadoption, abandonment, scale-up, spread, and sustainability) framework [21,22].

**Table 1:** Theoretical acceptance and adoption models.

Model	Developer	Year	Theoretical basis	Central constructs	Points of critique	Extended models
TAM^a^	Davis [9,11,12]	1985	Cognitive psychology [13]	Describes elements to predict the degree to which a person plans to perform specific future behavior. It suggests that perceived usefulness, perceived ease of use, and attitude (ie, intention to use) can explain user motivation [12]. It is a way to predict the intended use of a technology.	Mainly conceptualized for the acceptance of individuals and is not useful for explaining acceptance of electronic health (eHealth) technologies by organizations [11,14].	TAM2 (by Venkatesh and Davis) and TAM3 (by Venkatesh and Bala) [9,12]
DOI^b^	Rogers [9]	1995	Diffusion research [18]	Explains the characteristics of innovation. Observability, trialability, complexity, relative advantage, and compatibility are the primary determinants of innovation diffusion, which help explain the different rates of adoption [9,15]. Diffusion starts with recognizing the user’s need. It spreads by knowledge acquisition, persuasion, decision (ie, adopt or reject), implementation (ie, routine use, reinvention, and conformation), promotion, and evaluation [15].	There is little focus on the organizational context [14,16,17].	The unifying theoretical model of Greenhalgh et al [16] and the Consolidated Framework for Implementation Research (CFIR) of Damschroder et al [17,18]
UTAUT^c^	Venkatesh et al [9,19]	2003	Cognitive psychology [13]	Builds on TAM and focuses on perceptions and assumptions of people, resulting in the intention to use technology. States that constructs like performance expectancy, effort expectancy, social influence, and facilitating conditions influence intention and ultimately behavior [19]. These four constructs are moderated by gender, age, experience, and voluntariness of use [19].	Does not deal with hindrances to actual use [13]. Excludes users’ cognitive, affective, and physical ability to use technology [20] and ignores technological factors that might influence the decision to use an application [20].	UTAUT2 by Venkatesh et al developed in 2012 [9]
NASSS^d^ framework	Greenhalgh et al [21,22]	2017	Complexity theory [21,22]	Points to aspects explaining the complexity of technological innovations in health care, which according to all the described models influence the adoption. Includes the value proposition (ie, supply-side and demand-side values) as an important factor, in contrast to many implementation theories that do not [14,21].	Not found yet	Not found yet

^a^TAM: Technology Acceptance Model.

^b^DOI: diffusion of innovations.

^c^UTAUT: Unified Theory of Acceptance and Use of Technology.

^d^NASSS: nonadoption, abandonment, scale-up, spread, and sustainability.

All four theoretical models include two key concepts—acceptance and adoption—which are either ill-defined or used interchangeably. The concept of acceptance focuses on if, how, and when intended users would use a technology [23], and adoption is the actual use of an eHealth technology. Different stages in an adoption process can result in the actual use of an eHealth technology [16]. During an adoption process, users of eHealth technology develop feelings about the technology, gain experience, find meaning or do not find meaning in its use, and evaluate the functions of the technology [16]. Thus, there is a difference between intended use (ie, acceptance) and actual use (ie, adoption) [13].

The complexity of the adoption process of eHealth technologies is underexposed in all four theoretical models. The literature reports that adoption is a highly complex process and that results cannot be *made* or even predicted [13,16]. Greenhalgh et al highlight specific prerequisites for each of the three adoption process stages for innovations [16]: (1) in the preadoption stage, intended adopters should know about the innovation: in this case, the patient portal, (2) in the early use stage, intended adopters should be supported in using the innovation and learn how to fit or blend it into their daily routines, (3) in the adoption stage, established users arise if they gain an understanding of the consequences of using of the innovation and if they have the opportunity to refine and improve it: in this case, a patient portal [16]. The theoretical models provide no clarity on the pragmatics of efforts in the adoption process. For instance, they do not show how hospitals can encourage patients and HCPs to adopt a patient portal. Therefore, we studied the introduction of patient portals in 12 Dutch hospitals, using a multi-actor perspective to gain a broad understanding of the experiences and views on adoption. Our empirical study focuses on the pragmatics of stimulating the adoption of a patient portal. This paper answers the following research question: How can hospitals encourage patients and HCPs to adopt a patient portal?

## Methods

### Design and Setting

In this qualitative study, we asked participants from various backgrounds how they encouraged users to adopt a patient portal and what they had learned from their efforts, in order to understand what stimulated or hindered the adoption process. We conducted 22 multi-actor, semistructured group interviews and held four structured focus groups to check, enlarge, and enrich our findings [24-26]. Table 2 lists the different forms of data collection.

**Table 2:** Data collection.

Type of data collection and participants		Number of participants (N=122), n (%)	Data collection moments (N=26), n (%)
**Individual and group interview**			
	Patients	22 (18.0)	22 (85)
	Health care professionals	16 (13.1)	22 (85)
	Organizational staff	31 (25.4)	22 (85)
**Focus group**			
	Project leaders and staff 1	14 (11.5)	2 (8)
	Project leaders and staff 2	28 (23.0)	2 (8)
	Patient portal suppliers	5 (4.1)	1 (4)
	Macro stakeholders	6 (4.9)	1 (4)

All interviews, both group and individual, and focus group sessions took place in a hospital or online. All the hospitals included in this study are participating in a national program—VIPP (Versnellingsprogramma Informatie- Uitwisseling Patiënt en Professional) [27]—initiated by the Dutch Hospital Association and the Dutch government. VIPP is the Dutch government’s financial incentive program to support information exchange between patients and professionals through patient portals. The aim of the VIPP program is to give patients online access to their medical data, either through a patient portal or a PHR [27]. Information technology (IT) suppliers with a commercial interest deliver patient portals and PHRs. Given that Dutch hospitals are free to choose any supplier for their patient portals or PHRs, the hospitals in this study use different patient portals. The portals might differ in their available functionalities, but they all offer patients online access to their personal health information [6]. Additionally, hospitals had different aims for their patient portals; only the VIPP program aims were similar for all Dutch hospitals. Hospitals receive financial support based on their achievement of specific national VIPP aims; for example, “In the past 30 days, 25% of all patients (based on DRG [diagnosis-related group] contacts) logged in to the patient portal or the link to a PHR” [28]. How hospitals achieve these aims is left up to the hospital.

### Recruitment and Participants

There are three categories of Dutch hospital: academic, teaching, and general. Academic hospitals were excluded from our study because at the time they were not participating in the VIPP program. We first determined inclusion criteria for general and teaching hospitals, aiming for a diverse study group. Based on user statistics of patient portals (low and high), geographical differences (rural areas and cities), variation in patient portal suppliers, and usage of the patient portal (more or less than one year), the researchers (PH, AMWJ, and BP) made a selection of targeted hospitals.

In total, 15 of 64 Dutch hospitals (23%) were approached, of which 12 (80%: 10 teaching and 2 general) agreed to participate. Reasons for not participating included “already participating in another study” (n=1) and “too busy with implementation and fear of not meeting VIPP deadlines,” which would mean losing financial support (n=2).

At each hospital or via the Zoom online platform [29], the researchers arranged individual and group interviews in close collaboration with the person running the implementation of the patient portal at that particular hospital. Most often, this person was the project leader who selected participants according to a predefined list of three roles:

1. Patients: patients and client council members (n=22).
2. HCPs: physicians, Chief Medical Information Officers (CMIOs), nurses, Chief Nursing Information Officers (CNIOs), pharmacists, and outpatient clinic staff (n=16).
3. Organizational staff: project leaders, project staff, communication advisors, legal policy makers, and managers (n=31).

In total, 69 participants were included (see Table 2) if they were older than 18 years and had experience with developing and/or using a patient portal. The researchers emailed invitations to participants of the individual and group interviews; groups ranged from 2 to 6 participants. In 10 of the participating hospitals, we organized one or two group interviews; in the remaining two hospitals we conducted one individual interview for logistical reasons.

The aim of the focus groups was to check, enlarge, and enrich our results. Two of the four focus groups were held with project leaders and project staff of hospitals. They joined one of two self-selected focus groups organized during an educational meeting of the VIPP program (n=14 and n=28, respectively). For the other two, we used targeted sampling, selecting experts from patient portal suppliers (n=5) for the third group. The fourth group included macro stakeholders: Ministry of Health, NICTIZ (Nationaal ICT Instituut in de Zorg), the center of expertise for eHealth, health insurance companies, and scientific experts (n=6). All focus group members were invited to take part by email.

### Data Collection and Analysis

Qualitative data were collected on-site in the hospital (n=59) or online via the Zoom platform [29] (n=10) in the fall of 2018. The Zoom platform enabled the inclusion of hard-to-reach, geographically dispersed participants [30]. Group interviews lasted an average of 72 minutes (range 53-88) and the individual interviews lasted an average of 53 minutes (range 44-65). One researcher (PH) conducted all the interviews, following a predefined topic list (see Multimedia Appendix 1) [31] that was based on a search of the literature and discussed among the research team.

The four focus groups lasted an average of 82 minutes (range 71-88) and were steered by a Microsoft PowerPoint presentation explaining the findings of our study. No revisions were made in the presentation between focus group sessions, ensuring that varying opinions were heard before conclusions were drawn [32]. Each focus group was run by two researchers (PH and BP or PH and AMWJ) complementing each other: one moderating and the other taking notes.

During both group interviews and focus group sessions we encouraged the exchange of heterogeneous views that provided insights into similarities and differences in the opinions and experiences of the various stakeholders [26,30,31]. We also invited the participants to challenge each other’s views [33], to explore the implications of their thinking, and to articulate their sometimes-implicit assumptions about the adoption process. This method generated new insights through group interactions.

We audio-recorded the on-site interviews and focus groups and video-recorded the online sessions. All interviews were transcribed verbatim. Analysis, comprising six phases [34], was not linear but a recursive process. First, each individual researcher gave the transcripts a close reading. Second, one researcher (PH) developed codes for the interesting parts of the data. Next, three researchers (PH, AMWJ, and BP) independently developed themes, reaching consensus on a list of relevant themes (eg, communication channel, ambassadors, and patient participation [35]) through discussion. Fourth, one researcher (PH) read the transcripts again. Fifth, using the list of themes, one researcher (PH) performed thematic coding, which two other researchers (AMWJ and BP) checked. In the final phase we (PH, AMWJ, and BP) started writing the results [34].

### Ethical Approval

The Medical Ethics Review Committee of Erasmus Medical Center (Erasmus MC) approved our research proposal (MET-2018-1531) and checked if we were General Data Protection Regulation (GDPR) compliant. All participants were asked to sign an informed consent form.

## Results

### Overview

Our study aims to provide insights into the adoption process of a patient portal by patients and HCPs. The efforts of hospitals to stimulate the use of patient portals can be categorized under three themes: (1) informing patients and professionals about the patient portal, (2) embedding the patient portal in the daily routine of HCPs, and (3) adjusting the portal to patients’ needs in the initial and continuous development process. Below we present our empirical findings for each of these themes.

### Informing Patients and Health Care Professionals About the Patient Portal

#### Overview

Participants agreed that communication about the patient portal is very important for the adoption of patient portals. The hospitals require the involvement of communication experts to inform patients and HCPs. We identified four objectives to support patient portal adoption using the informing of patients and HCPs as the basis. Here we explain them one by one. Participants mentioned using 23 communication channels to reach their audiences (see Table 3).

**Table 3:** Channels (N=23) used to achieve four communication objectives to inform patients and health care professionals.

Channel	Knowing about the portal	Knowing how the portal works	Knowing that action is required on the portal	Knowing where to find help with the portal
Pocket tickets	x^a^			
Promotion leaflets	x			
Press releases	x	x		
Posters	x			
Banners	x			
Screen in waiting room	x	x		
Social media	x			
Video on website	x	x		
Explanatory leaflets	x	x		
Students or volunteers in central hall for a week	x	x		x
Physical point with employees and volunteers	x	x		x
Health care professionals	x	x	x	x
Letter with request (before or after appointment)			x	
Text message with request			x	
Mail with request			x	
Staffed desk				xxx
Informative meetings	xx			
Interactive meetings	xxx	xxx		
Internal website	xx			
Peer to peer	xx	xx	xx	xx
Training	xx	xx	xx	
Newsletter	xx			
Goodie bag with explanation	xx	xx		

^a^Key: x = patients; xx = professionals; and xxx = patients and professionals.

#### Objective 1: Knowing About the Portal

First, participants argued that it is obviously necessary to make sure that patients and HCPs know about the patient portal; otherwise, they cannot use it. Meeting this objective means that patients and HCPs will have a general idea of what the portal is and what it looks like. The hospitals used various mass-focused channels most frequently to communicate the existence of the portal to patients and HCPs. These channels include leaflets and posters, banners hung in the corridors, messages on social media, and placing volunteers in the central hall of the hospital to point patients to the portal. Using multiple channels to reach patients is considered important because patients have different preferences, but it is probably a bit inefficient, as these quotes illustrate:

I am not inclined to take leaflets from the hospital. If it’s really important, I think I will be reminded.

Participant #0.4, client council member

All those freebies [goodie bags], I don’t like them. They won’t get me to look at the portal.

Participant #14.2, patient, informal caregiver, and former client council member

Still, it works for others.

Participant #11.4, client council member

Looking at the channels used to reach HCPs, we see hospitals organizing informative presentations for medical and nursing staff and department meetings. Participants felt that informing only the team leaders and managers is not enough; it is important to directly inform the HCPs. According to participants, effective channels that reach broad groups of professionals include department meetings, the hospital intranet, and the staff newsletter. Reaching out by email is considered inefficient because HCPs receive a lot of email and particular messages can be easily missed or skipped.

#### Objective 2: Knowing How the Portal Works

Second, participants argued that patients and professionals need to know how the patient portal works. Hospitals can meet this objective by (1) using video screens in waiting rooms, (2) putting explanatory videos on the hospital website, and (3) placing volunteers in the central hall of the hospital to teach patients how the portal works.

Hospitals asked professionals who are already successful portal users to explain how the portal works to their peers (ie, peer-to-peer information). One hospital gave their professionals a goodie bag with explanatory flyers during portal training, but only a few hospitals organized training sessions on new functionalities for HCPs. However, the participants said that HCPs need to know how the portal actually works as patients sometimes turn to them for help with portal questions:

So often I hear that people can’t log in to the patient portal. I think that as staff we should be looking into the portal far more. How does it actually work for the patient in practice?

Participant #1.2, staff member

If the HCP has to tell the patient that they do not know how to help, this can be a disappointment. One participant reflected the following:

Lots of patients say, “I'm logged in but then I don't know [what to do].” Many colleagues say, “I don't know either.” If you can’t help the patient, they’ll drop out immediately.

Participant #5.5, senior doctor’s assistant

#### Objective 3: Knowing That Action Is Required on the Portal

A third objective is to entice patients and HCPs to visit the portal. Hospitals did this by sending specific calls to action, including personal letters, text messages, or emails asking the recipient to read through an online brochure on their treatment before their hospital appointment or to fill in a questionnaire:

Your appointment letter also asks you to complete the pre-operative screening questionnaire at home.

Participant #9.1, communication advisor

Hospitals encouraged HCPs to use the patient portal by asking them to respond to e-consults (ie, messages sent by the patient) and by showing them functions that will save their time or make their work more efficient. Time-saving functions like preoperative screening questionnaires on the patient portal are easy to embed in the daily routine of professionals. One participant explained the following:

We built the pre-operative screening questionnaire in such a way that [the information the patient provides] gets entered directly into the anesthesiologist's outpatient file. [This means] that the moment the anesthesiologist starts the consultation with the patient, the data are already in the system. The outpatient clinic started using the questionnaire right from the go-live.

Participant #12.1, project leader

#### Objective 4: Knowing Where to Find Help With the Portal

The fourth communication objective is to ensure that patients and HCPs know where to look for help when they have a problem using the portal. Hospitals organized a help desk, publishing its phone number and email address in leaflets, letters, on the hospital website, and on the portal itself. Some hospitals organized a service point clearly visible in the central hall of the hospital, where patients receive face-to-face service. Outpatient clinic staff can tell patients about this service point. HCPs can ask their colleagues for help (ie, peers).

Reviewing these hospital communication strategies, three findings are worth a mention:

1. The texts used to inform patients and HCPs have a promotional tone. Hospitals choose to stress the benefits and hardly mention the potential disadvantages or risks of portal use.
2. Mass communication is preferred because it is less labor intensive. However, it is also less personal and less in line with the needs of an individual. Personal communications, such as letters, text messages, and emails, have the advantage in that they probably make patients and health care professionals feel personally addressed.
3. Hospitals struggle with the timing of starting their communication efforts, for example, having a silent “go-live” or starting a campaign directly after launching the portal.

Our participants explained that most hospitals do not inform patients about the go-live, because this gives them the opportunity to solve start-up problems and technical issues signaled by the first users. After some two to three months they will use a cross-media approach to communicate on the portal. Our participants expressed concern that if a hospital initiated a communication campaign straight after launching the portal, the hospital could be making promises that they cannot keep.

### Embedding the Patient Portal in Daily Routines

#### Overview

In the previous section we showed how communication strategies support patient portal adoption processes. HCPs worry that using the portal is time-consuming and will disturb their daily routine. They regard explaining how the portal works and communicating with patients on the portal as extra tasks and expect that helping non-computer-literate patients (eg, the elderly and people with low literacy) will be especially time-consuming. One participant explained the following:

If a patient says, “I don’t use the computer,” I won’t ask if they have a son or daughter who’d like to share their access. I don’t start with that, it costs too much time, really.

Participant #5.5, senior doctor’s assistant

Our data show three ways to support embedding the patient portal in the daily routine of HCPs and management: (1) hospital policy, (2) management by monitoring the numbers, and (3) a structured implementation strategy that includes all employees in the department, termed a specialism-focused approach. According to our participants, all three ways require changing work processes and routines.

#### 1: Hospital Policy

The first way to embed patient portals is by developing hospital policy on digitalization. Our participants revealed that some hospitals lack hospital-wide agreements, resulting in a lack of coherence for the patient. One such hospital-wide agreement could set the maximum time that HCPs have to respond to an e-consult. According to our participants, the CMIO and the CNIO can play an important role in setting hospital-wide agreements and explaining new work routines to their colleagues (ie, peer influence). Our participants also said that some hospitals set no obligation or targets to use the portal:

How can we make sure the patient portal lands properly in the outpatient clinic? Good question... Well, it may have landed, but there is still no obligation [to use it] and that’s the real problem.

Participant #1.1, communication advisor

For example, it is not clear within which time frame HCPs need to respond to patients’ e-consults or how many patients per specialism should be using the portal. Responses to patients should be prompt, and the professionals need time to incorporate their responses into their daily work processes on the portal. One participant explained the following:

If you have to explain something to the patient on the portal or send them an email within four hours, then we need to think about how to get that complex planning process in the system. The professionals need time to think about it too. So that's development; and you need even more time to implement.

Participant #13.2, CNIO

#### 2: Management by Monitoring the Numbers

The second way to embed patient portals is by ensuring that management monitors information on portal use or response time. Most hospitals lack management control of portal use. Some hospitals, however, monitor the numbers of new patient accounts and users on a dashboard or monitoring system. In one hospital, outpatient clinic employees must ask all patients presenting themselves at the desk if they are interested in opening a patient portal account. Hospital management uses this monitoring information to talk with staff who do not seem to be encouraging enough patients to sign up for the portal. One participant revealed the following:

Staff must register whether or not they have asked if a patient is interested in having a portal account. Now we can run reports on the employee level... We do that sometimes and then we can see that, say, Marie scores 100% on “not interested.” Of course, ... then you’d have to start the conversation.

Participant #5.4, care and operations manager

Our participants said that fear is possibly a reason why HCPs do not motivate patients to sign up for the portal. Professionals need to overcome their own unfamiliarity and prejudices by experiencing the benefits of the patient portal. One participant declared the following:

Of course, we do it for the patient, but let's see where it helps the physician. Then you’ll get them to at least use the patient portal.

Participant #8.7, internist

#### 3: Specialism-Focused Approach

A third way to embed portals in daily routines is to apply a specialism-focused approach—a structured implementation strategy—that includes all the staff in the department. This involves a multidisciplinary project team (eg, communication advisor and project leader), management, and HCPs temporarily collaborating on changing work processes to benefit the incorporation of the patient portal into daily routines. Not trying to convince just one physician, but the whole department (eg, the outpatient clinic team), makes it easier to embed the patient portal. Working closely with project staff gives the HCPs support that is based on their needs or wishes. This approach requires giving HCPs the time to discuss their problems, share their experiences, and experiment. One participant explained the following:

Using this approach, we’ve really looked in depth at the points where the portal can be embedded better in their work process. For example, we’ve supported the specialism of rheumatology. They have very clear ideas about using the portal. Now we’ll work actively with the health care professionals in the coming period to increase the use of the patient portal within their specialism.

Participant #11.2, communication advisor

According to our participants, using this approach supports giving professionals an understanding of how the portal works and how they can use it in their daily routines. However, they said that it is labor intensive for everyone involved, which slows down the adoption process hospital-wide.

Another implementation strategy is for hospitals to start off the portal adoption processes with keen, intrinsically motivated HCPs. Hospitals put effort into these professionals. They are seen as ambassadors, as *game changers*, who will convince other HCPs by setting a good example. One participant said the following:

I believe that starting out with the enthusiastic specialists is the most successful strategy and that’s why we’re starting with people who want it. We’re not setting out with the difficult ones who don’t want it.

Participant #11.3, CMIO

### Adjusting the Portal to Meet Patients’ Needs

#### Overview

Our participants said that in the continuous development process, adjusting the portal to meet the patients’ needs is important. For example, enhancing user-friendliness ensures repeated use of a portal. As well, asking patients for feedback on the portal can reveal points of improvement that the project staff might not spot, as our participants explained:

An example: a patient tests the portal, first on a dummy and later on their own file. Someone remarks: “It’s in chronological order, but the most recent is at the bottom. Why don’t you put the most recent at the top of the page?”

Participant #4.2, project leader

It’s as simple as that. You don’t notice that when you are so involved.

Participant #4.1, advisor of functional management

According to our participants, another reason they find it important to adjust to patients’ needs is because the perspectives of the patient and the communication advisor may differ:

I’m against all those abbreviations... Why not explain what they are? I guarantee you that half the patients won’t know what the abbreviation means. Add an abbreviation list.

Participant #10.6, patient and client council member

It’s my choice. I can write the term in full, I can explain it the abbreviation. But when I write it out completely, it becomes a very long sentence.

Participant #10.2, communication advisor

Our participants mentioned two methods hospitals use in the effort to optimize portal user-friendliness: (1) patient feedback and (2) focus on optimizations for patients with special needs (eg, low literacy, visually impaired, and low digital skills).

#### 1: Patient Feedback

The first way to adjust to patients’ needs in the continuous development of patient portals is to set up a panel of patients to act as a sounding board or to survey patients on their experiences and wishes. One participant reported the following:

We have a panel of 150 people. We sent these people a questionnaire on the patient portal and how they would like to use it.

Participant #3.2, client council member

Another way is to organize sessions with patients to test portal functionalities (eg, access to data, an e-consult, and filling in questionnaires). To illustrate, one hospital organized a test session for feedback and observations:

We invited a few patients from our patient panel. We gave them a test version of the portal and asked them to do a few assignments and fill in a questionnaire. For example: look at the patient portal and see if you get it. Give as much feedback as possible about the things that could be improved... There was one-on-one guidance. We had a large number of employees involved, so that we could sit next to the patients and get as much feedback as possible. So, we could also see how things went.

Participant #7.1, project employee

Hospitals also asked for feedback and reused questions, comments, and complaints patients express to the helpdesk. One participant said the following:

We now actively request feedback from patients. The helpdesk also receives feedback and phone calls and we can use the input obtained.

Participant #1.1, communication advisor

#### 2: Focus on Optimizations for Patients With Special Needs

A second way to adjust to patients’ needs is by optimizing the portal for people with special needs. For example, language experts check the language used on the portal and written information on how to use it, removing jargon and abbreviations and simplifying texts for patients with low literacy. They make more use of visuals (eg, icons, pictograms, and infographics):

You can summarize in pictograms, which makes it much easier for patients with low literacy. Visuals work better and faster.

Participant #9.2, patient

Another example of optimizing portal use for people with special needs is when hospitals collaborate with organizations offering general computer courses, such as the municipality, community centers, and libraries, for patients with few digital skills. Hospitals ask those organizations to blend the patient portal into their course and teach patients to work with it. Also, hospitals may refer patients to this course if they do not have computer skills and need to learn how to work with the Dutch national identity authentication method (DigiD). Participants report that the DigiD is not easy to use and its log-in process requires many steps:

I find the accessibility of the patient portal a real problem. Logging in with your DigiD is difficult.

Participant #3.3, functional manager

We look for courses on using the DigiD subsidized by the municipality. They organize courses in the community centers for people having trouble with DigiD and then these people can practice logging in on the patient portals.

Participant #11.2, communication advisor

Despite the importance of adjusting to patients’ needs in continuous development, hospitals sometimes hesitate to include patients, because they may not be able to act on the patients’ feedback. For example, if patients miss functionalities, it can require time and money to add them to the portal and, therefore, this cannot be easily fixed. Participants mentioned the importance of explaining to patients what the hospital does with their feedback and why some feedback points cannot be solved in the near future (eg, technologically impossible or too expensive). Otherwise, patients will feel that the hospital is not taking their feedback seriously. The following quote shows how hospitals struggle to let patients participate in the continuous portal development, even though they find patient input invaluable:

If we invite the panel group for testing, then we have to show that we have improved the portal based on their feedback... Otherwise they will think “nothing happens with our input.” If we organize patient participation, you can only say “we’re too busy” once.

Participant #4.1, advisor of functional management, and participant #4.2, project leader

One of the challenges of acting on patient feedback stems from the collaboration with the suppliers of patient portals. Suppliers will undertake to improve or develop new functionalities when multiple hospitals make the same request. Surprisingly, however, suppliers (n=5) said that they include no patients in their development process. The suppliers see it as the responsibility of their customers—the hospitals—to give voice to patients’ wishes.

## Discussion

### Principal Findings

This qualitative study focuses on patient portal adoption processes by patients and HCPs in a Dutch hospital context. Overall, our results show that the adoption of patient portals is more dynamic than presented in theoretical models and the literature. Greenhalgh et al’s linear adoption stages (ie, preadoption, early use, and established users) [16] seem useful in studying adoption by individuals, but hospital patients and HCPs are in different adoption stages. Consequently, an organization cannot simply move through sequential stages; it needs ongoing effort to be put into informing, embedding, and adjusting to patients’ needs. Their focus on individuals rather than the organizational context is also a criticism levied at the theoretical models (see Table 1) [14,16,17].

All participating hospitals seem to be experimenting with stimulating adoption of the patient portal. They are trying to create effective communication strategies, looking for the best way to embed the portal in daily routines and adjust to its patients’ needs. As yet, they have not found the best way of encouraging portal use by patients and HCPs. Here we explain the implications of our results.

Our study shows that hospitals are experimenting with many communication channels (N=23), mostly ones that are already in use. Despite efforts by communication departments, it seems that portal adoption is still quite a challenge. It seems that hospitals do not know which channels are most effective for which target audiences and what the right timing is for their communication campaigns. Looking at their communication strategies, we found that hospitals choose to emphasize the benefits of portal use and hardly mention the potential disadvantages or risks. According to Greenhalgh et al [16], intended adopters must know the consequences of adopting a patient portal to become established users. If intended adopters are not informed of the potential disadvantages, then they cannot oversee all the consequences of using the portal, for example, the risks. However, the financial incentives of the VIPP program may explain the positive promotion strategy. If hospitals do not attain a certain adoption percentage (ie, 10% or 25%) they will have to repay their VIPP grant.

Our results show that hospitals invest in HCP adoption through peer-to-peer influence. However, focusing on the enthusiastic HCPs can mean that the less-motivated HCPs will lag behind. That a patient portal often does not reduce the burden of HCPs (ie, it only means extra work) and that it is not embedded properly in work routines can hinder adoption. The specialism-focused approach offers a way of encouraging patient portal adoption by HCPs. This experiment with portal embedding would be interesting to study in other contexts to see where and how it could lead to better embedding of the patient portal. A possible disadvantage of this approach could be that patients will not understand why specialisms are in different adoption stages (ie, patients can make an online appointment with one specialism but not for another).

Another principal finding is that hospitals are struggling to adjust the portal to meet patients’ needs in their continuous development process, although all seem to find this important. According to Greenhalgh et al [16], it is vital that intended users get the opportunity to refine the portal so that they will not drop out in the early adoption stage. Hospitals are using various ways to adjust to the patients’ needs in ongoing portal development, without knowing which one is most effective in which phase and for what purpose. It would be interesting to do more research on how patients can participate in portal development, including efforts to stimulate adoption.

Nonusers of patient portals could be studied further. Previous studies show that nonusers have various reasons for not adopting the portal [36,37], including a preference to speak directly to their HCP, the level of their communication skills [36], and their concern for privacy and information security [36]. Such studies would show that hospitals are taking nonusers’ concerns seriously and, at the same time, could produce insights valuable to exploring whether and how the patient portal could be made useful to them.

A remarkable finding is that portal suppliers do not include patients in their development process. The suppliers see it as their clients’ responsibility to give voice to patients’ wishes, but the focus group discussion did make them rethink this. This means that hospitals must explain to the supplier how they should make the portal more user-friendly for patients. Because of the variation in hospital context and portal suppliers, this could explain the disappointing adoption by patients.

### Limitations

Our study has four limitations. The first is that the adoption processes in the hospitals we studied might be somewhat unusual due to the financial incentives of the national VIPP program. Conducting similar research in other countries would, therefore, be interesting and could also teach us more about the contextual, including cultural, factors that influence hospitals’ efforts to stimulate adoption.

The second limitation is the way we recruited hospital participants. Using our own research networks may have biased our sample. However, our recruiting process resulted in a good variation in the mix of included hospitals.

The third limitation is that we only included teaching and general hospitals, given that academic hospitals follow another implementation program. Also, during the study period they were not participating in the VIPP program and, therefore, could not be compared. However, the inclusion criteria context of the studied hospitals varied greatly to include different kinds of hospitals and patients.

Last, this descriptive study shows the efforts that some Dutch hospitals have made to stimulate adoption of a patient portal. We did not study whether the undertaken efforts led to an actual increase of the adoption of patient portals. A further study on the effectiveness of these efforts is recommended.

### Comparison With Prior Work

In recent years, many theoretical models on the adoption of information technology have been developed [9,10]. These models show which variables are important for the adoption of a technology; for example, *perceived ease of use*, defined in the Technology Acceptance Model (TAM) as “the degree to which the person believes that using the particular system would be free of effort” [12]. However, these models are not explanatory and do not provide the know-how to stimulate patient portal adoption [9,10]. Consequently, we suggest future research should not focus on models, including new ones, but should deal with actionable knowledge for practice [38]. Action research can be used to study the adoption process and the embedding of patient portals in daily practice [39].

Communication experts support the hospitals’ choice to use a cross-media promotion to inform patients and HCPs about the patient portal [40]. Explaining the benefits of using a patient portal is especially important for promotional messages [41]. However, open dialogue among HCPs and project leaders and staff is also vital because it illuminates the professionals’ perspectives on portal development [41]. Earlier research shows that ignoring doubts while trying to convince others to use a technology may produce negative energy and a reluctance to use the portal [41,42]. Further research is required to find the most effective hospital communication strategies for encouraging patient portal adoption for patients and HCPs.

Our study showed that embedding a patient portal in the daily routine of HCPs requires changing their work processes. Earlier studies suggest that hospitals need to make extra time available to HCPs so that they can change and learn new work processes [41]. Research shows that portal use by patients may increase when HCPs are active on the patient portal and it is embedded in their work processes [43]. Research suggests training can benefit the adoption process [41], yet only some hospitals organize courses that explain how the portal works. As a result, some HCPs lack familiarity with portal functionalities [42].

The literature reports several ways of using feedback to adjust the portal to patients’ needs in the continuous development of patient portals. These studies could help hospitals struggling with this. It is important to include patients at the beginning of ongoing development of patient portals [1,44,45]. Examples include co-design, where patients help identify the project based on personal experiences in collaborating with the clinician [45], and participatory stakeholder co-design, where patients and clinicians are equal stakeholders in the whole project [45]. Vulnerable patient groups, such as disadvantaged older adults, should be given special attention in the process of cocreation and user testing [7]. This is an important issue for future research.

### Conclusions

Patient portal adoption processes are not just about implementing the technology. They require human interaction in a multitude of ways. Our study reveals three key findings for the adoption process: (1) informing patients and HCPs about the portal, (2) embedding it in the daily routine of HCPs, and (3) adjusting it to patients’ needs in the continuous development of the portal. Our paper provides rich insights into the complexity of the adoption process and gives examples of efforts to stimulate the adoption of patient portals. Our findings help to translate the relatively abstract factors mentioned in the theoretical models to the everyday pragmatics of eHealth projects in hospitals.

## Appendix

Multimedia Appendix 1 Topic list for interviews.

## Abbreviations

CMIO: Chief Medical Information Officer
CNIO: Chief Nursing Information Officer
DigiD: Dutch national identity authentication method
DRG: diagnosis-related group
GDPR: General Data Protection Regulation
eHealth: electronic health
EHR: electronic health record
Erasmus MC: Erasmus Medical Center
HCP: health care professional
NASSS: nonadoption, abandonment, scale-up, spread, and sustainability
NICTIZ: Nationaal ICT Instituut in de Zorg
PHR: personal health record
TAM: Technology Acceptance Model
VIPP: Versnellingsprogramma Informatie-Uitwisseling Patiënt en Professional
