# Supplementary material for: Adopting Patient Portals in Hospitals: Qualitative Study
Source: J Med Internet Res. 2020 May 19;22(5):e16921. doi: 10.2196/16921 (PMC7268003; doi:10.2196/16921)
Supplement: Multimedia Appendix 1 [file jmir_v22i5e16921_app1.docx]

| Topic list |  |  |
| --- | --- | --- |
| Setting/context | All participants | Introduce yourself |
|  | Project leader | - When did the hospital introduce the patient portal? - Description patient portal - Functionalities? - Who can use it? - Aims - Intended effects? - Target usage percentages? |
|  | Patient | - When did you first use the patient portal? - Motivation? - Extent of use? - Reasons for use? - Experienced advantages? - Example? - Experienced disadvantages? - Example? - When do you think you will use the patient portal again? Why? |
|  | Project manager | - User statistics available? - Why available, why not? - How often are statistics collected? - What is done with the statistics? - Audit report? |
|  | Healthcare professional | - Evaluation   - Research done?   - Positive experiences?   - Negative experiences?   - How is feedback collected? |
| **Open question** | All participants | - Promoting use of patient portal? - Example per activity - Limitations for use of patient portal? - Example per limitation |
| **Focused interventions, based on factors derived from the literature** | Patient | Communication to you   - Actions? - Resources? - Examples? - What works well? - What is missing? |
|  | Healthcare professional | What are your experiences with communication about the patient portal? |
|  | Project manager | What are your experiences with communication about the patient portal? |
|  | Patient | What can you use the patient portal for?   - Experiences? - Example(s)? |
|  | Project manager | How do you manage the differences in skills among patients?   - Experiences? - Example(s)? |
|  | Patient | What do you want to use the patient portal for?   - Experiences? - Example(s)? |
|  | Healthcare professional | How do you manage the differences in patients’ needs/wishes?   - Experiences? - Example(s)? |
|  | Patient | How easy/hard do you think it is to use the patient portal (scale 1-10)   - Do you understand everything shown in the patient portal? - What do you do if something does not work? - What do you do when you do not understand? - Example(s)? |
|  | Project manager | Is there specific attention for patients with low literacy?   - Example(s)? |
|  | Patient | “Training” for you?   - Why (not)? - What kind of training was it? - Effect(s) of training? - Not: would you like training? |
|  | Project manager | “Training” for patients?   - Why (not)? - What kind of training? - Effect(s) of training? - Have you heard any patients’ experiences? |
|  | Patient | Guidance in presented information (written documentation)?   - How? - Example(s) |
|  | Project manager | Guidance in presented information?   - How? - Example(s)? |
|  | Patient | Staffed (help) desk?   - What works and what does not work? - Experiences? - Example(s)? |
|  | Healthcare professional | Helpdesk?   - What works and what do not work? - Experiences? - Example(s)? |
|  | Project manager | Helpdesk?   - How is it managed? - Who staffs the help desk? - What works and what does not work? |
|  | Healthcare professional | Communication to you   - Actions? - Resources? - Example(s)? |
|  | Project manager | Communication to healthcare professional   - Actions? - Resources? - Example(s)? |
|  | Healthcare professional | “Training” for you?   - Why (not)? - What kind of training was it? - Experienced effects? |
|  | Project manager | “Training” for healthcare professional?   - Why (not)? - What kind of training was it? - Experienced effects? |
|  | Patient | Encouraged by physician / healthcare professional   - Yes/No? - Why (not)? - How? - Example(s) |
|  | Healthcare professional | Giving encouragement to patiens   - Yes/No? - Why (not)? - How? - Example(s)? |
|  | Healthcare professional | What is your experience of working with the patient portal?  How have your work processes changed by using the patient portal? |
|  | Patient | Have you authorized anyone to look (share) in your patient portal?   - Yes/No? - Why (not)? - Experience(s)? |
|  | Project manager | Shared access   - Experience(s)? |
|  | All participants | User-friendliness   - Experience(s)? - Example(s)? |
| **Technological factors** | Patient | Focusing purely on the patient portal   - What works well? - What could be improved? - What does the patient portal lack? |
|  | Healthcare professional | Technical/content-based aspects  What works well in the patient portal?  What does not work well? |
|  | Project manager | Technical/content-based aspects  What works well in the patient portal?  What does not work well? |
| **Last question** | All participants | Imagine the hospital gets 2 thousand euro for improving the patient portal. What would you recommend? What should the hospital spend the money on?   - This could be one or more things |
| **End** | Healthcare professional or project leader | Are there any healthcare professionals or patients open to joining a focus group in your organization? |
|  | All participants | Is there anything else you would like to tell us? |
